# Outcome of manual thrombus aspiration for patients undergoing primary PCI for acute STEMI showing large thrombus burden

**DOI:** 10.1186/s43044-020-00122-9

**Published:** 2021-01-12

**Authors:** Ehab Mohamed Elfekky, Michael Nabil Penjameen, Ahmed Ibrahim Nassar, Ramy Raymond Elias

**Affiliations:** Department of Cardiology, Faculty of Medicine, Ain Shams University, Abbasia, Cairo, Egypt

**Keywords:** STEMI, Thrombus aspiration, TIMI flow, ST-segment resolution, MBG

## Abstract

**Background:**

Routine thrombus aspiration during primary PCI does not improve clinical outcomes. Although recent guidelines for management of patient presented by ST-elevation myocardial infarction treated by primary PCI does not recommend routine use of thrombus aspiration and ranking it as class III recommendation. However, there is remaining uncertainty about rule of TA in group of patients presented by STEMI and their initial coronary angiogram shows large thrombus burden as there is a logical rationale for greater benefit, and still, there are no clear guidelines for management of this group of patients; the aim of this study is to evaluate the in-hospital and short-term outcome of using manual thrombus aspiration in STEMI patients undergoing primary PCI and showing large thrombus burden.

**Results:**

The study was prospective observational study included 209 patients referred to coronary care unit (CCU) with diagnosis of STEMI who had undergone primary PCI; their initial coronary angiography show totally occluded infarct-related artery with heavy thrombus burden. Patients were divided into 2 groups: group (B) which included 73 patients, who had undergone PTCA and manual thrombus aspiration followed by stent to culprit lesion, and group (A) which included 136 patients, who had undergone conventional PTCA and stent of culprit lesion within the period from November 2016 till July 2018. Patients had a pre-discharge echo and were followed up for 4 weeks for major adverse cardiac events (MACE) and echo repeated after 1 month of discharge.

It was found that thrombus aspiration in heavy thrombus burden cases had improved in-hospital mortality and in-hospital secondary endpoints (TIMI flow, MBG, ST-segment resolution), as well as 30-day MACE and LV systolic function.

**Conclusion:**

In patients presented with STEMI and heavy thrombus burden culprit artery, manual thrombus aspiration has great value in reducing index hospitalization and 1 month mortality and improve TIMI flow, MBG, ST-segment resolution, and left ventricular systolic function.

## Background

Primary percutaneous coronary intervention (PPCI) is a gold standard treatment for patients with ST-segment elevation myocardial infarction (STEMI) [1, 2]. PPCI is superior to fibrinolysis in improving epicedial flow and myocardial reperfusion and myocardial blush grade with more patency of the infarct-related artery, faster and more complete resolution of ST-segment elevation. In aggregate, these benefits translate into reduced infarct size and improved survival [3]. Fibrinolysis has higher rate of reocclusion and reinfection [4].

Value of thrombus aspiration (TA) depends on decreasing distal embolization and protection of microcirculation, which frequently occurs in patients presenting with ST-elevation myocardial infarction (STEMI) treated with primary percutaneous coronary intervention (PCI) [3, 5–11]. In spite of its appealing conceptual value, there are conflicting data yielded in randomized clinical trials may be due to differences in clinical presentation of patients included in different studies and heterogeneity in angiographic characteristics mainly inclusion of both occluded and patent culprit vessels and different grade of thrombus burden at the time of the initial coronary angiography [12–24].

Results of routine thrombus aspiration during primary PCI was disappointing and did not improve clinical outcomes. However, there is remaining uncertainty about the potential benefit in those patients with high thrombus burden, where there is a biological rationale for greater benefit [25].

### Aim of study

The aim of this study is to evaluate the in-hospital and short-term outcome of using manual thrombus aspiration in STEMI patients undergoing primary PCI and showing large thrombus burden.

## Methods

We enrolled 209 patients referred to coronary care unit within 12 h of chest pain with diagnosis of STEMI patients; their initial coronary angiography show totally occluded infarct-related artery with heavy thrombus burden. Patients were divided into two groups based on whether thrombus aspiration was attempted. This decision was left at the operator’s discretion.

Patients were divided into 2 groups: group (B) which included 73 patients, who had under-gone PTCA and manual thrombus aspiration followed by stent to culprit lesion, and group (A) which included 136 patients, who had undergone conventional PTCA and stent of culprit lesion within the period from November 2016 till July 2018. Patients had echocardiography on day 1 of MI and were followed up after 4 weeks for SWMA, estimated EF by Simpson technique; patients were followed up for 4 weeks for major adverse cardiac events (MACE). Patients with small thrombus burden were excluded from the study. All selected patients have been subjected to complete history taking, full clinical examination, Lab workup, including CBC, kidney function test, and cardiac biomarkers. Resting 12 lead ECG and full echocardiographic study. All the patients received acetylsalicylic acid, P2Y12 inhibitors, and UFH during the procedure. During the procedure, the culprit vessel was determined and if there was any other vessel involvement either for intervention in the same session or later on. Also, the thrombus grading scale as well as TIMI flow and MBG were evaluated pre- and post-PCI. For patients assigned to thrombus aspiration, guidewire placement was followed by Diver C thrombus aspiration catheter then stent placement at culprit lesion.

The Diver CE (Invatec, Brescia, Italy) is a rapid-exchange, 6-F compatible, and thrombus-aspirating catheter. It has a central aspiration lumen and a soft, flexible, 0.026-inch, non-traumatic tip with multiple holes (one large anterior and three smaller lateral ones) communicating with the central lumen. A 30-ml lower lock syringe is connected to the proximal hub of the central lumen for thrombus-aspiration [26].

### Analytical statistics

1. Chi-square test was used to examine the relationship between two qualitative variables but when the expected count is less than 5 in more than 20% of the cells; Fisher’s exact test was used.
2. Independent sample *t* test was used to assess the statistical significance of the difference of a parametric variable between two independent means of two study groups.

*P* value: Level of significance

- *P* > 0.05: non significant (NS)
- *P* < 0.05: significant (S)
- *P* < 0.01: highly significant (HS)

## Results

1—Baseline clinical features (Table 1)

**Table 1:** Comparison between 2 groups regarding clinical features

	Thrombus aspiration		Test value	*P* value	Sig.
	No (A)	Yes (B)			
	No. = 136	No. = 73			
Age (years)					
Mean ± SD	55.76 ± 10.3	55.00 ± 11.58	0.490	0.625	NS
Range	27-88	24-76			
Gender					
Females	28 (20.6%)	13 (17.8%)	0.233	0.629	NS
Males	108 (79.4%)	60 (82.2%)			
BW					
Mean ± SD	86.99 ± 7.51	86.85 ± 6.74	0.129	0.897	NS
Range	60-100	70-100			
BMI					
Mean ± SD	30.1 ± 2.60	30.1 ± 2.33	0.129	0.897	NS
Range	20.7-34.6	24.22-34.6			
DM					
Positive	67 (49.3%)	28 (38.4%)	2.280	0.131	NS
HTN					
Positive	68 (50.0%)	37 (50.7%)	0.009	0.925	NS
Smoking					
Positive	99 (72.8%)	54 (74.0%)	0.034	0.854	NS
Hashish smoking					
	126 (92.6%)	63 (86.3%)	2.210	0.137	NS
Positive	10 (7.4%)	10 (13.7%)			
Obesity					
Positive	70 (51.5%)	37 (50.7%)	0.012	0.914	NS
Renal impairment					
Positive	23 (16.9%)	17 (23.3%)	1.248	0.264	NS
FH of IHD					
Positive	22 (16.2%)	16 (21.9%)	1.053	0.305	NS
PH of MI					
Positive	5 (3.7%)	5 (6.8%)	1.050	0.306	NS
PH of PCI					
Positive	10 (7.4%)	4 (5.5%)	0.267	0.606	NS
PH of CABG					
Positive	1 (0.7%)	0 (0.0%)	0.539	0.463	NS
History of angina					
Positive	34 (25.2%)	16 (21.9%)	0.277	0.599	NS

2—Location of myocardial infarction (Table 2)

**Table 2:** Comparison between 2 groups regarding type of MI prevalent in each group

	Thrombus aspiration		Test value	*P* value	Sig.
	No	Yes			
	No. = 136	No. = 73			
Anterior	74 (54.4%)	39 (53.4%)	6.854	0.232	NS
Inferior	30 (22.1%)	12 (16.4%)			
Lateral	4 (2.9%)	0 (0.0%)			
Posterior	0 (0.0%)	1 (1.4%)			
Anterolateral	3 (2.2%)	4 (5.5%)			
Inferoposterior	25 (18.4%)	17 (23.3%)			

**Fig. 1:**
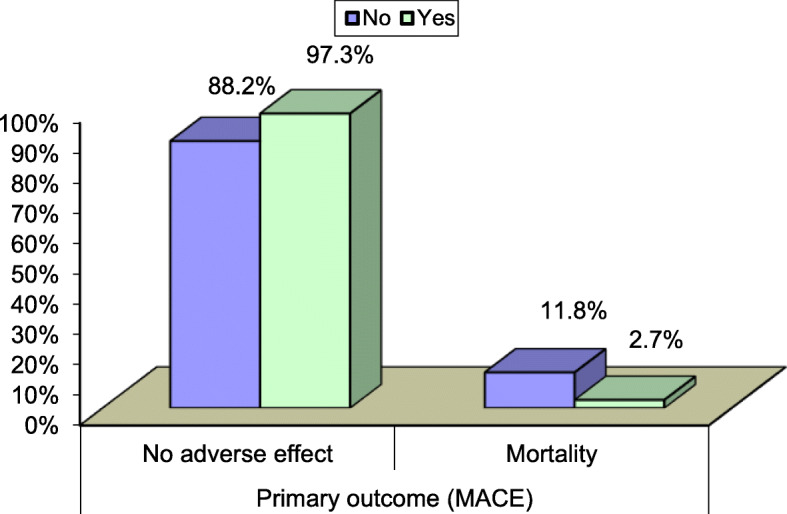
Mortality benefit among thrombus aspiration group

3—Type of used stent (Table 3) (Fig. 1)

**Table 3:** Comparison between the 2 groups regarding intervention, the number of stent needed in thrombus aspiration group was significantly less than the conventional group

Stent type	Thrombus aspiration		Test value	*P* value	Sig.
	No	Yes			
	No. = 136	No. = 73			
No	5 (3.7%)	10 (13.7%)	7.162	0.007	HS
BMS	112 (82.4%)	52 (71.2%)	3.477	0.062	NS
DES	18 (13.2%)	9 (12.3%)	0.035	0.852	NS
CABG	1 (0.7%)	2 (2.7%)	1.349	0.245	NS

**Fig. 2:**
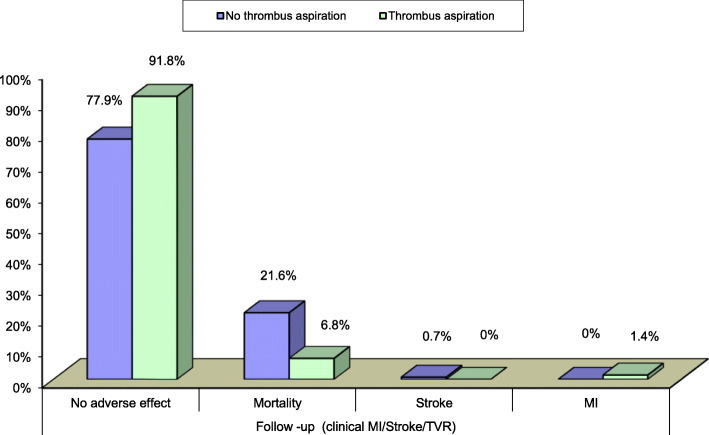
Statistical significant difference between the 2 groups regarding MACE at 1 month follow-up

4—Relation between the 2 groups, regarding primary endpoints MACE (mortality, non-fatal MI, stroke, target vessel revascularization) (Table 4) (Fig. 2)

**Table 4:** Mortality was significantly lower in the thrombus aspiration group that had occurred in 2.7% of group (B), but in 11.8% of group (A). There was non-significant difference between the two groups as regard other complication (stroke, non-fatal MI, and target vessel revascularization)

	Thrombus aspiration		Test value	*P* value	Sig.
	No	Yes			
	No. = 136	No. = 73			
Peri-procedural arrhythmic complications					
No	130 (95.6%)	68 (93.2%)	3.766	0.43	NS
Bradyarrythmia	1 (0.7%)	0 (0.0%)			
V. Tachycardia	4 (2.9%)	4 (5.5%)			
Atrial fibrillation	1 (0.7%)	0 (0.0%)			
Arrest (hypoxic)	0 (0.0%)	1 (1.4%)			
Peri-procedural vascular complications					
No	129 (94.9%)	69 (94.5%)	0.011	0.91	NS
Yes	7 (5.1%)	4 (5.5%)			
Primary outcome (MACE)					
No adverse effect	120 (88.2%)	71 (97.3%)	4.916	0.02	S
Mortality	16 (11.8%)	2 (2.7%)

5—Comparison between the 2 groups, regarding secondary end-points (Table 5)

**Table 5:** Comparison between the 2 groups regarding TIMI flow, MBG, ST-segment resolution

	Thrombus aspiration		Test value	*P* value	Sig.
	No	Yes			
	No. = 136	No. = 73			
TIMI					
I	9 (6.6%)	1 (1.4%)	7.823	0.020	S
II	18 (13.2%)	3 (4.1%)			
III	109 (80.1%)	69 (94.5%)			
MBG					
0	10 (7.4%)	1 (1.4%)	14.121	0.003	HS
I	6 (4.4%)	0 (0.0%)			
II	41 (30.1%)	12 (16.4%)			
III	79 (58.1%)	60 (82.2%)			
ST-segment resolution					
< 70%	18 (13.2%)	2 (2.7%)	16.674	0.000	HS
> 70%	118 (86.8%)	71 (97.2%)			

There was a statistical significant difference between 2 groups regarding TIMI flow, MBG, and ST-segment resolution. TIMI III flow had been achieved in 94.5% of group (B) but had been achieved in 80.1% of group (A). MBG 3 had been achieved in 82.2% of group (B) but had been achieved in 58.1% of group (A). ST-segment resolution > 70% had been achieved in 97.2% of group (B) that had been achieved in 86.8% in group (A).

6—Comparison between the two groups, regarding follow-up after 1 month: There were statistical significant results regarding clinical follow-up, while no adverse outcome had developed in 91.8% of group (B), no adverse outcome had developed in 77.9% of group (A). Mortality had occurred in 6.8% of group (B), but in 21.6% of group (A). Stroke had occurred in 1 case of group (A). Non-fatal MI had occurred in 1 case of group (B).

7—Comparison between the 2 groups, regarding follow-up echocardiography data: Improvement in left ventricular systolic function detected in day 1 of MI and after 1 month follow-up, there were highly significant results in both groups (A) and (B). In group (B), LVEF% had improved from 42.08%+/−8.7 to 51.68%+/−9.5, but had improved from 41.57%+/−9.34 to 46.74%+/−9.38 in group (A) (Table 6).

**Table 6:** Statistical significant results regarding improvement of LV systolic function in both 2 groups

	Admission results	Follow-up results	Difference	Test value	*P* value	Sig.
No thrombus aspiration						
Mean ± SD	41.57 ± 9.34	46.74 ± 9.38	5.17 ± 0.65	4.952	< 0.001	HS
Range	21-65	28-67				
Thrombus aspiration						
Mean ± SD	42.08 ± 8.70	51.68 ± 9.50	9.6 ± 1.05	8.24	< 0.001	HS
Range	27-62	28-68				

## Discussion

Management of STEMI has been developed over the last years and primary percutaneous coronary intervention, where available, has become the therapy of choice. However, there are some obstacles to achieve successful myocardial reperfusion. One of the most famous obstacles is high thrombus burden which is present in a significant proportion of patients, wire manipulation and balloon dilatation and stent placement in culprit vessel with high thrombus burden cause distal embolization of thrombus particles and atherosclerotic plaque debris impaired myocardial perfusion resulting in poorer short- and long-term outcomes, including heart failure and death.

Management of culprit lesions with high thrombus burden including both pharmacological and mechanical measures to decrease distal embolization and microvascular obstruction these measures includes pharmacological measures, such as adequate antiplatelet therapy, glycoprotein IIb/IIIa antagonists, and coronary vasodilators as adenosine, adrenalin, and verapamil, mechanical evacuation of thrombus as devices dedicated to evacuating or trapping thrombus during intervention to reduce the risk of distal embolization during percutaneous coronary intervention.

One of the most appealing devices dedicated to evacuate thrombi from culprit artery is thrombus aspiration (TA) devices which have been proposed as adjunctive therapy to protect microcirculation from distal embolization [3, 11].

Thrombus aspiration has appealing conceptual value but also conflicting results yielded in randomized clinical trials so this therapy receives only a class of recommendation IIa, level of evidence B according to European Guidelines 2008 [24], and class III recommendation in European Guidelines 2017.

A number of small-scale or single-center studies and one meta-analysis of 11 small trials suggested that there could be benefits from routine manual thrombus aspiration during primary PCI [27].

One of the earlier trials is REMEDIA trial which was published in JACC 2005; this trial randomized 100 consecutive patients with STEMI to either standard PCI or PCI with manual thrombus aspiration. Primary endpoints of the study were ST-segment resolution (STR) ≥ 70% and post-procedural rates of myocardial blush grade (MBG) ≥ 2 and which were respectively, 44.9% and 68.0% in the thrombus-aspiration group compared with 36.7% and 58.0% in the standard PCI group. Moreover, the rate of patients achieving both the angiographic and electrocardiographic (ECG) criteria of optimal reperfusion was significantly higher in the thrombus-aspiration group compared with standard PCI: 46.0% versus 24.5% [28].

TAPAS trial which was a relatively large randomized trial; it was published in 2008 randomized 1071 STEMI patients assigned for primary PCI to the thrombus-aspiration group or the conventional-PCI group before undergoing coronary angiography. A myocardial blush grade of 0 or 1 occurred in 26.3% of the patients in the conventional-PCI group and in 17.1% of those in thrombus-aspiration group (*P* < 0.001). Complete resolution of ST-segment elevation occurred in 44.2% and 56.6% of patients, respectively (*P* < 0.001). At 30 days, the rate of death in patients with a myocardial blush grade of 0 or 1, 2, and 3 was 5.2%, 2.9%, and 1.0%, respectively (*P* = 0.003), and the rate of adverse events was 14.1%, 8.8%, and 4.2%, respectively (*P* < 0.001) [22].

Recently, two large randomized controlled trials > 7000 patients, which were adequately powered to detect value of routine manual thrombus aspiration in PPCI versus conventional primary PCI procedure and influence the guidelines recommendation, unfortunately, both trials showed no benefit on clinical outcomes of routine aspiration strategy. Also, there are safety concerns that emerged in the trial of routine aspiration thrombectomy with PCI versus PCI alone in patients with STEMI (TOTAL) trial (*n* = 10 732), with an increase in the risk of stroke [29].

We suppose these disappointing results may be yielded by two main causes, the first cause is heterogeneity of patients and wide scale of thrombus burden and heterogeneity of coronary anatomy, second cause is concept of routine use of thrombus aspiration. So we think if we limiting the use of thrombus aspiration device to patients with totally occluded culprit artery by large thrombus burden, we may get the expected benefits of this technique.

In the subgroup of TOTAL trial with high thrombus burden [TIMI (thrombolysis in myocardial infarction) thrombus grade 3], thrombus aspiration was associated with fewer cardiovascular deaths [170 (2.5%) vs. 205 (3.1%); hazard ratio (HR) 0.80, 95% confidence interval (CI) 0.65–0.98; *P* = 0.03] and with more strokes or transient ischemic attacks [55 (0.9%) vs. 34 (0.5%); odds ratio 1.56, 95% CI 1.02–2.42, *P* = 0.04]. However, the interaction *P* values were 0.32 and 0.34, respectively.

In the taste and TOTAL trials, 1–5% of randomized patients crossed over from PCI alone to thrombus aspiration. Based on these data and the results of a recent meta-analysis, the last European guidelines for the management of STEMI patients which was published in 2017 stated that routine thrombus aspiration is not recommended, but in cases of large residual thrombus burden after opening the vessel with a guidewire or a balloon, thrombus aspiration may be considered [30].

In our study, we tried to evaluate the rule of thrombus aspiration therapy in improving the outcome of primary PCI in STEMI patients with high thrombus burden. Patients were divided into 2 groups: group (B) which included 73 patients, who had undergone PTCA and manual thrombus aspiration followed by stent to culprit lesion when needed, and group (A) which included 136 patients, who had undergone conventional PTCA and stent of culprit lesion within the period from November 2016 till July 2018.

There was no significant difference between both groups as regard clinical background or location of myocardial infarction. The number of needed stents in the thrombus aspiration group is significantly less than conventional PCI group.

Cardiovascular mortality was significantly lower in the thrombus aspiration group that had occurred in 2.7% of patients, but in 11.8% of conventional PCI group. There was a non-significant difference between the two groups as regard other complication (stroke, non-fatal MI, and target vessel revascularization).

There was a statistical significant difference between 2 groups regarding TIMI flow, MBG, ST-segment resolution. TIMI III flow had been achieved in 94.5% of the thrombus aspiration group but had been achieved in 80.1% of conventional PCI group. MBG III had been achieved in 82.2% of the thrombus aspiration group but had been achieved in 58.1% of the conventional PCI group. ST-segment resolution > 70% had been achieved in 97.2% of thrombus aspiration group that had been achieved in 86.8% in conventional PCI group.

After 1 month of primary PCI, while no adverse outcome had developed in 91.8% of the thrombus aspiration group and 77.9% of conventional PCI group. Mortality had occurred in 6.8% of thrombus aspiration group significantly less than conventional PCI group (21.6%). Stroke had occurred in 1 case of conventional PCI group. Non-fatal MI had occurred in 1 case of the thrombus aspiration group.

## Conclusion

Our trial concluded that thrombus aspiration therapy improves outcome of primary PCI in STEMI patient with high thrombus burden without significant increase in the incidence of stroke and must be considered in selected patients with high thrombus burden.

## Data Availability

The datasets used and analyzed during the current study are available from the corresponding author on reasonable request.

## Abbreviations

PCI: Percutaneous coronary intervention
PTCA: Percutaneous transluminal coronary intervention
STEMI: ST elevation myocardial infarction
CCU: Coronary care unit
TIMI flow: Thrombolysis in myocardial infarction flow
MBG: Myocardial blush grade
TA: Thrombus aspiration
SWMA: Segmental wall motion abnormality
CBC: Complete blood picture
UFH: Unfractionated heparin
BW: Body weight
BMI: Body mass index
DM: Diabetes mellitus
HTN: Hypertension
IHD: Ischemic heart disease
MI: Myocardial infarction
BMS: Bare metal stent
DES: Drug-eluting stent
